# Primary hyperparathyroidism unmasking after dienogest-altered estrogen dynamics: implications for calcium homeostasis

**DOI:** 10.1210/jcemcr/luag188

**Published:** 2026-07-13

**Authors:** Hiroyuki Yamashita, Daisuke Tatsushima, Hisakazu Shindo, Yusuke Mori, Kento Katsuyama, Shinya Sato

**Affiliations:** Department of Surgery, Yamashita Thyroid Hospital, Fukuoka City, Fukuoka 812-0034, Japan; Department of Surgery, Yamashita Thyroid Hospital, Fukuoka City, Fukuoka 812-0034, Japan; Department of Surgery, Yamashita Thyroid Hospital, Fukuoka City, Fukuoka 812-0034, Japan; Department of Surgery, Yamashita Thyroid Hospital, Fukuoka City, Fukuoka 812-0034, Japan; Department of Surgery, Yamashita Thyroid Hospital, Fukuoka City, Fukuoka 812-0034, Japan; Department of Surgery, Yamashita Thyroid Hospital, Fukuoka City, Fukuoka 812-0034, Japan

**Keywords:** primary hyperparathyroidism, normocalcemic primary hyperparathyroidism, estrogen, menopause, progestin therapy

## Abstract

Normocalcemic primary hyperparathyroidism (PHPT) is increasingly recognized as an early or indeterminate disease form. Although estrogen is believed to modulate calcium homeostasis, its role in progression to overt PHPT remains incompletely understood. A parathyroid adenoma was suspected in a 33-year-old woman despite normal calcium and parathyroid hormone (PTH) levels. At 37 years of age, approximately 6 months after starting dienogest 0.5 mg twice daily for menorrhagia, she developed prolonged amenorrhea and improved anemia. The PTH level subsequently increased from 38 to 57 pg/mL (SI: 4.0-6.0 pmol/L) (reference range 10-65 pg/mL [SI: 1.1-6.9 pmol/L]) with mild hypercalcemia (corrected calcium, 10.3 mg/dL [SI: 2.58 mmol/L]) (reference range, 8.8-10.1 mg/dL [SI: 2.20-2.53 mmol/L]). Despite the prolonged amenorrhea, her estradiol levels were not suppressed. The dienogest was discontinued preoperatively and menstruation resumed; however, the hypercalcemia and elevated PTH persisted. Imaging revealed the parathyroid adenoma. Upon its resection, the PTH level promptly normalized. Altered estrogen dynamics rather than an absolute estrogen deficiency may have contributed to the transition to overt PHPT noted herein. Manifested hypercalcemia is not readily reversible, suggesting a shift to a self-sustaining state. This case raises the possibility that altered hormonal dynamics may contribute to the manifestation of PHPT.

## Introduction

Primary hyperparathyroidism (PHPT) is a common endocrine disorder characterized by autonomous parathyroid hormone (PTH) secretion and hypercalcemia. Normocalcemic PHPT has increasingly been recognized as an early or indeterminate form of the disease in which PTH levels are elevated despite normal serum calcium concentrations [1-3]. Although this phenotype is considered a potential precursor of overt PHPT, the factors that drive its progression to hypercalcemia remain poorly understood. Primary hyperparathyroidism is more frequently diagnosed in postmenopausal women [3], suggesting a role of hormonal influences, including estrogen deficiency. Estrogen modulates calcium homeostasis through its effects on skeletal remodeling and the calcium–PTH relationship [4-6].

In clinical practice, parathyroid lesions identified on imaging in patients with normal biochemical findings are often considered nonfunctioning or incidental. Conversely, elevated PTH levels may be detected in normocalcemic individuals during the evaluation for conditions such as nephrolithiasis or fragility fractures [1]. In both scenarios, the subsequent development of overt hypercalcemia raises important questions regarding disease evolution and potential triggers of biochemical manifestations. However, longitudinal clinical observations linking such transitions to changes in estrogen dynamics are scarce.

Here, we report a case of PHPT that progressed from a normocalcemic or indeterminate state to overt hypercalcemia associated with altered estrogen dynamics during dienogest therapy. This case highlights the potential role of hormonal dynamics in modulating the calcium–PTH relationship and unmasking the underlying parathyroid disease.

## Case presentation

A 33-year-old woman was referred for the evaluation of a thyroid nodule. Ultrasonography revealed a benign-appearing nodule in the left thyroid lobe and a well-defined hypoechoic lesion posterior to the lower pole of the right thyroid lobe suggestive of a parathyroid tumor. At that time, serum corrected calcium (cCa) and intact PTH levels were within the normal range (cCa, 9.7 mg/dL [SI: 2.43 mmol/L]; reference range, 8.8-10.1 mg/dL [SI: 2.20-2.53 mmol/L]; PTH, 31 pg/mL [SI: 3.3 pmol/L]; reference range, 15-65 pg/mL [SI: 1.6-6.9 pmol/L]). In the absence of biochemical abnormalities, the lesion was considered clinically nonfunctional or biochemically indeterminate, and the patient was followed up with periodic observation.

During follow-up, serum cCa and PTH levels remained within the normal range. The patient had chronic anemia, with hemoglobin levels consistently below 11 g/dL (SI: 110 g/L) (reference range, 11.3-15.2 g/dL [SI: 113-152 g/L]), which was attributed to menorrhagia.

## Diagnostic assessment

Approximately 4.5 years later, mild hypercalcemia (cCa, 10.5 mg/dL [SI: 2.63 mmol/L]) with a nonsuppressed intact PTH level (52 pg/mL [SI: 5.5 pmol/L]) was detected, and the maximum diameter of the parathyroid tumor had increased from 11.2 mm at the initial visit to 14.9 mm. Her hemoglobin level had normalized (14.4 g/dL). A further clinical inquiry revealed that the patient had started progestin therapy (dienogest 0.5 mg twice daily) 6 months earlier for dysmenorrhea that had resulted in amenorrhea. Despite prolonged amenorrhea, the serum estradiol and follicle-stimulating hormone levels were 68 pg/mL (SI: 250 pmol/L) and 8.5 mIU/mL (SI: 8.5 IU/L), respectively. The dienogest was discontinued, and menstruation resumed with 2 cycles before parathyroidectomy; however, the hypercalcemia and elevated PTH levels persisted during this interval.

The patient's serum phosphate levels were within the normal range at initial presentation and during the first year of follow-up, but gradually decreased thereafter. They reached 2.5 mg/dL (SI: 0.81 mmol/L) (reference range, 2.5-4.5 mg/dL [SI: 0.81-1.45 mmol/L]) in the second year and remained at 2.3-2.4 mg/dL (SI: 0.74-0.78 mmol/L) throughout the remainder of the follow-up period, including after the initiation of dienogest therapy and the subsequent emergence of hypercalcemia.

Her serum 25-hydroxyvitamin D level was 7.2 ng/mL (SI: 18 nmol/L) (reference range, >20 ng/mL [SI: >50 nmol/L]). This parameter was measured only once before surgery, and no vitamin D supplementation had been administered before or during dienogest therapy. The bone-specific alkaline phosphatase level was 13.2 μg/L (SI: 13.2 μg/L) (reference range, 2.9-14.5 μg/L), while the tartrate-resistant acid phosphatase 5b (TRACP-5b) level was 469 mU/dL (SI: 46.9 U/L) (reference range, 120-420 mU/dL [SI: 12-42 U/L]) during dienogest therapy. Approximately 2 months after discontinuation of dienogest, serum estradiol levels increased from 68 to 233 pg/mL (SI: 250-855 pmol/L), while TRACP-5b decreased to 323 mU/dL (SI: 32.3 U/L). Serum cCa levels remained unchanged at 10.3 mg/dL (SI: 2.58 mmol/L), whereas intact PTH levels increased slightly from 52 to 57 pg/mL (SI: 5.5-6.1 pmol/L).

Her bone mineral density was preserved according to dual-energy X-ray absorptiometry (lumbar spine T-score, −0.4; femoral neck, +0.7; total radius, +1.2). The patient's renal function was preserved.

**Figure 1:**
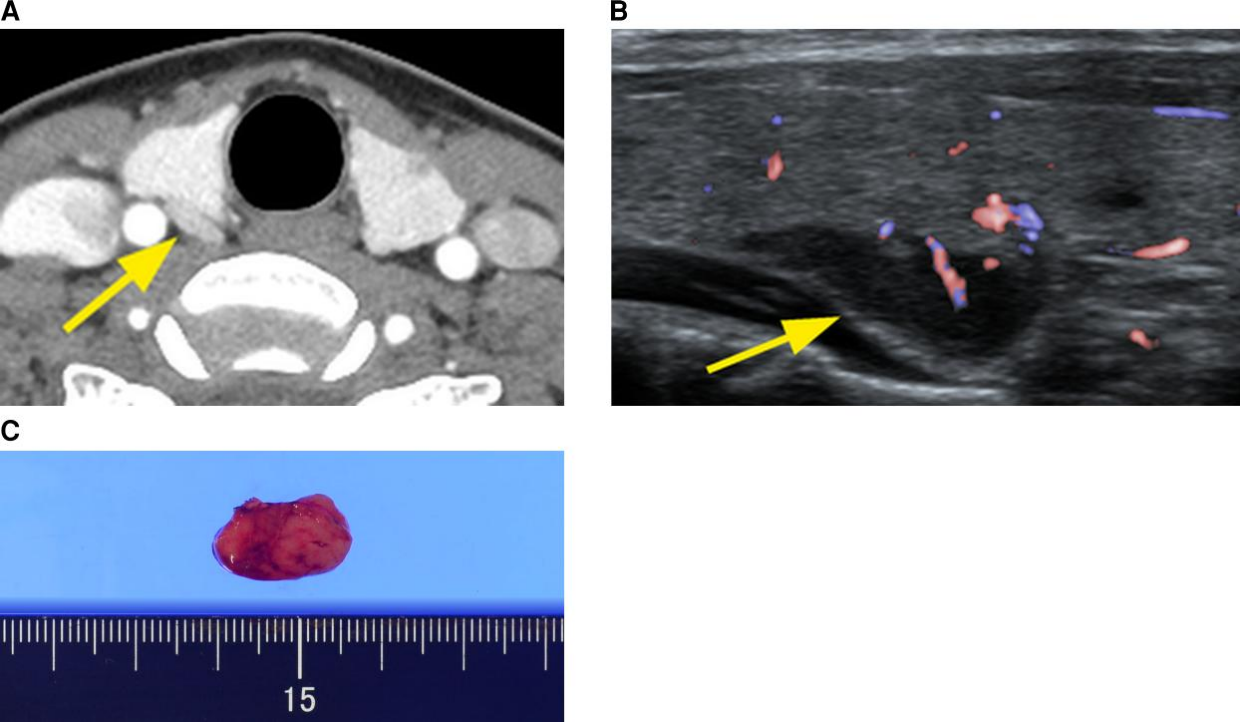
Imaging and macroscopic findings of the 550-mg parathyroid lesion. (A) Four-dimensional computed tomography image showing a corresponding lesion (arrow). (B) Ultrasonography image demonstrating a well-defined hypoechoic lesion posterior to the lower pole of the right thyroid lobe (arrow) with internal vascularity. (C) Gross resected parathyroid adenoma (550 mg).

Four-dimensional computed tomography demonstrated findings consistent with a parathyroid adenoma and corresponding to the lesion previously identified on ultrasonography (Fig. 1A and 1B).

## Treatment

The patient underwent surgical resection of the right lower parathyroid gland. The excised gland weighed 550 mg and was histologically confirmed as a parathyroid adenoma (Fig. 1C). Normal parathyroid glands generally weigh approximately 30 to 50 mg.

## Outcome and follow-up

**Figure 2:**
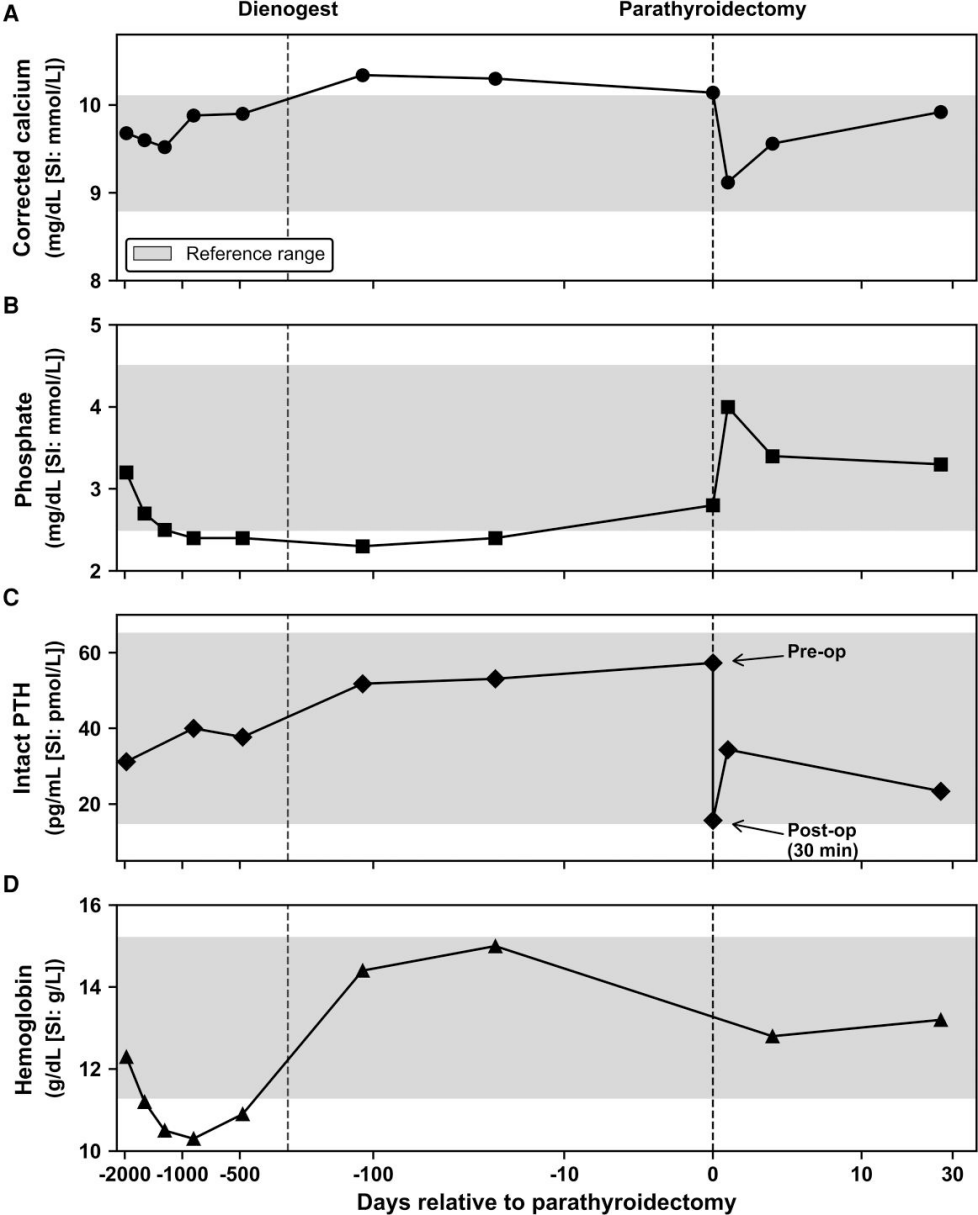
Longitudinal changes in biochemical parameters during the clinical course. (A) Serum cCa levels. (B) Serum phosphate levels. (C) Intact PTH levels. (D) Hemoglobin levels. The initiation of dienogest therapy, normalization of anemia, onset of hypercalcemia, and timing of parathyroidectomy are indicated. Note the transition from a biochemically indeterminate state to overt hypercalcemia following hormonal modulation, as well as the prompt decline in PTH levels after surgical resection. The x-axis is displayed on a compressed, nonlinear scale to illustrate both long-term follow-up and short-term postoperative changes. Negative values indicate the preoperative period. PTH, parathyroid hormone.

Baseline intraoperative PTH was 57 pg/mL (SI: 6.1 pmol/L) and decreased to 16 pg/mL (SI: 1.7 pmol/L) 30 minutes after excision according to our institutional intraoperative PTH monitoring protocol. The postoperative serum cCa levels normalized without complications. Figure 2 summarizes the longitudinal biochemical changes.

## Discussion

Primary hyperparathyroidism is more frequently diagnosed in postmenopausal women, and estrogen deficiency has long been hypothesized to unmask hypercalcemia in individuals with underlying parathyroid disease [6]. However, direct clinical documentation of such transitions remains limited, likely due to difficulty capturing longitudinal biochemical changes across hormonal states in routine practice.

This case demonstrates progression from a biochemically indeterminate state to overt PHPT in association with altered estrogen dynamics. At presentation, normal calcium and PTH levels masked functional disease despite the presence of a parathyroid lesion. Hypercalcemia emerged during dienogest therapy, possibly reflecting disruption of hormonal cyclicity rather than absolute estradiol suppression. Although estradiol levels were not in the hypoestrogenic range, prolonged amenorrhea suggests suppression of physiological hormonal cyclicity, which may have influenced calcium–PTH regulation.

An additional notable feature of this case is the persistence of hypercalcemia after discontinuation of dienogest. Although incomplete hormonal recovery cannot be fully excluded, the resumption of menstrual cycles and increase in estradiol levels suggest substantial restoration of hypothalamic–pituitary–ovarian axis activity. Moreover, TRACP-5b levels decreased after discontinuation of dienogest, indicating reduced bone resorptive activity, whereas hypercalcemia persisted and PTH levels increased slightly. These findings raise the possibility that, once hypercalcemia becomes manifest, the calcium–PTH regulatory system may transition to a self-sustaining state that is not readily reversible by normalization of hormonal conditions. This interpretation is further supported by the presence of a 550-mg parathyroid adenoma and the prompt postoperative decline in PTH, confirming that the biochemical abnormalities were attributable to a preexisting hyperfunctioning lesion rather than de novo disease. The relatively modest elevation in preoperative PTH is consistent with a transition from a compensated or indeterminate state to overt PHPT.

A key concept in interpreting this case is that the calcium–PTH relationship is not fixed but can be modulated by hormonal dynamics. Physiological studies have shown that estrogen shifts the calcium–PTH set point, allowing the suppression of PTH at lower calcium concentrations [4]. Conversely, alterations in the estrogen milieu may shift this relationship and facilitate the transition from a normocalcemic to overt phenotype [4, 5]. In the present case, hypercalcemia emerged in the absence of marked hypoestrogenemia, suggesting that disruption of the hormonal dynamics, rather than an absolute estrogen deficiency, may have contributed to this shift.

The gradual decline in serum phosphate levels prior to dienogest therapy suggests that the adenoma had already acquired biological activity through PTH-mediated renal phosphate wasting. Notably, serum phosphate levels remained largely unchanged after initiation of dienogest therapy despite the subsequent development of hypercalcemia. This observation suggests that the renal phosphaturic effect of PTH was already established before the hormonal intervention. In contrast, estrogen may modulate the renal phosphaturic and calcium-mobilizing skeletal effects of PTH differently. While estrogen may attenuate PTH-mediated skeletal calcium mobilization, it is less likely to directly inhibit PTH-induced renal phosphate wasting. Therefore, the presence of hypophosphatemia before dienogest therapy, together with the relative stability of serum phosphate levels thereafter, does not contradict the possibility that altered estrogen dynamics contributed to the later manifestation of hypercalcemia.

Conceptual parallels can be drawn with thiazide- or lithium-associated hypercalcemia, in which external factors alter calcium homeostasis and may unmask underlying parathyroid dysfunction [7, 8]. However, unlike these conditions, the present case involved a preexisting parathyroid adenoma that had not yet manifested as an overt biochemical disease. Therefore, the dienogest therapy appeared to have modified the physiological context in which the lesion became clinically apparent rather than inducing de novo PHPT.

Despite the widespread use of estrogen-suppressive therapies in patients with breast cancer [9], longitudinally documented cases of unmasking PHPT remain rare. This likely reflects the challenges in recognizing latent disease before overt biochemical abnormalities emerge as well as competing clinical explanations for hypercalcemia. Notably, despite efforts at our institution to identify similar cases following our previous report on normocalcemic PHPT [6], no clearly comparable cases were found. These observations suggest that such presentations may be under-recognized rather than truly uncommon.

This study had several limitations. First, the estradiol levels were assessed at limited time points, precluding a detailed evaluation of hormonal dynamics. Additionally, bone turnover markers and bone mineral density were not assessed before initiation of the dienogest therapy. Therefore, the proposed mechanism remained inferential.

In summary, this case suggests that alterations in estrogen dynamics, rather than absolute estrogen deficiency alone, may play a key role in the transition from a biochemically indeterminate state to overt PHPT. Furthermore, once hypercalcemia manifests, the biochemical state may persist despite the restoration of hormonal conditions, raising the possibility of a transition to a self-sustaining phenotype.

## Learning points

- Alterations in estrogen dynamics may facilitate the transition from a normocalcemic or biochemically indeterminate state to overt PHPT.
- The calcium–PTH relationship is modifiable and may shift in response to changes in hormonal dynamics rather than absolute estrogen levels alone.
- Once hypercalcemia manifests, it may persist despite withdrawal of the triggering factor, which reflects the underlying autonomous PTH secretion.
- External physiological or pharmacological factors may reveal the underlying rather than de novo induction of parathyroid disease.

## Contributors

All of the authors made individual contributions to authorship. H.Y., D.T., H.S., K.K., and Y.M. diagnosed and managed the patient. H.Y. performed the surgery. S.S. assisted with the patient's surgical management and contributed to the clinical interpretation of the case. All authors contributed to interpreting the findings, critically revising the manuscript for important intellectual content, and approving the final version.

## Data Availability

Data sharing is not applicable to this article as no datasets were generated or analyzed during the current study.
